# Comparative Transcriptome Reconstruction of Four *Hypericum* Species Focused on Hypericin Biosynthesis

**DOI:** 10.3389/fpls.2016.01039

**Published:** 2016-07-13

**Authors:** Miroslav Soták, Odeta Czeranková, Daniel Klein, Zuzana Jurčacková, Ling Li, Eva Čellárová

**Affiliations:** ^1^Department of Genetics, Institute of Biology and Ecology, Faculty of Science, Pavol Jozef Šafárik UniversityKošice, Slovakia; ^2^Institute of Mathematics, Faculty of Science, Pavol Jozef Šafárik UniversityKošice, Slovakia; ^3^Department of Genetics, Development, and Cell Biology, Iowa State University, AmesIA, USA; ^4^Center for Metabolic Biology, Iowa State University, AmesIA, USA

**Keywords:** *Hypericum* spp., RNA-Seq, *de novo* assembly, differential expression analysis, hypericin

## Abstract

Next generation sequencing technology rapidly developed research applications in the field of plant functional genomics. Several *Hypericum* spp. with an aim to generate and enhance gene annotations especially for genes coding the enzymes supposedly included in biosynthesis of valuable bioactive compounds were analyzed. The first *de novo* transcriptome profiling of *Hypericum annulatum* Moris, *H. tomentosum* L., *H. kalmianum* L., and *H. androsaemum* L. leaves cultivated *in vitro* was accomplished. All four species with only limited genomic information were selected on the basis of differences in ability to synthesize hypericins and presence of dark nodules accumulating these metabolites with purpose to enrich genomic background of *Hypericum* spp. *H. annulatum* was chosen because of high number of the dark nodules and high content of hypericin. *H. tomentosum* leaves are typical for the presence of only 1–2 dark nodules localized in the apical part. Both *H. kalmianum* and *H. androsaemum* lack hypericin and have no dark nodules. Four separated datasets of the pair-end reads were gathered and used for *de novo* assembly by Trinity program. Assembled transcriptomes were annotated to the public databases Swiss-Prot and non-redundant protein database (NCBI-nr). Gene ontology analysis was performed. Differences of expression levels in the marginal tissues with dark nodules and inner part of leaves lacking these nodules indicate a potential genetic background for hypericin formation as the presumed site of hypericin biosynthesis is in the cells adjacent to these structures. Altogether 165 contigs in *H. annulatum* and 100 contigs in *H. tomentosum* were detected as significantly differentially expressed (*P* < 0.05) and upregulated in the leaf rim tissues containing the dark nodules. The new sequences homologous to octaketide synthase and enzymes catalyzing phenolic oxidative coupling reactions indispensable for hypericin biosynthesis were discovered. The presented transcriptomic sequence data will improve current knowledge about the selected *Hypericum* spp. with proposed relation to hypericin biosynthesis and will provide a useful resource of genomic information for consequential studies in the field of functional genomics, proteomics and metabolomics.

## Introduction

**FIGURE 1:**
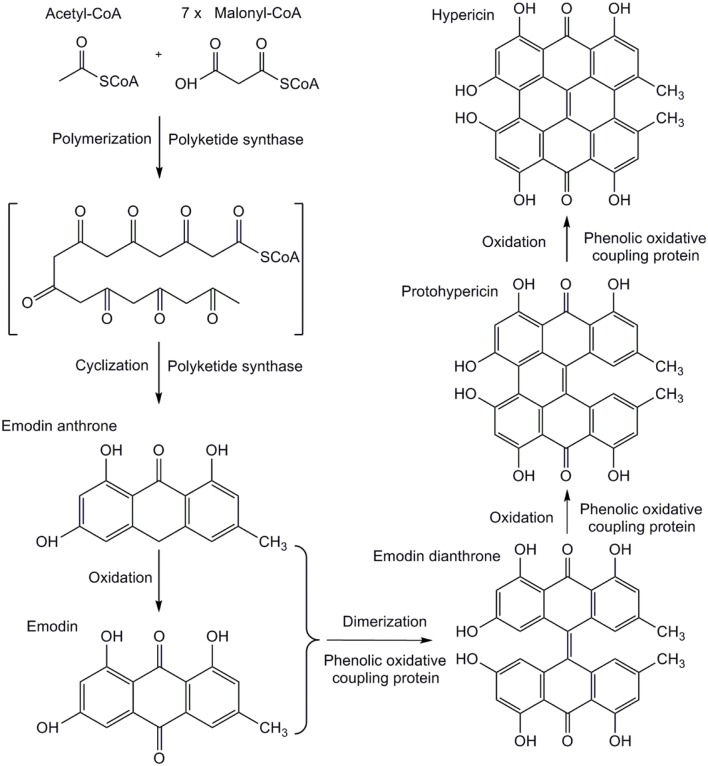
**Proposed biosynthetic pathway of hypericin**.

*Hypericum* is the genus with 496 species of plants spread worldwide (Nürk et al., 2013). The most of them are typical for compounds with anti-cancer (Agostinis et al., 2002), antioxidant (Silva et al., 2005), anti-viral (Birt et al., 2009), and anti-depressive (Butterweck, 2003) properties. Dark nodules (glands), the sites of hypericin accumulation are characteristic for approximately 2/3 of the taxonomic sections and are limited to particular organs (Robson, 2003). The metabolome of leaf tissue samples of *ex vitro* grown plants from the proximity to the dark nodules in *Hypericum perforatum* containing hypericin was visualized by the use of matrix-assisted laser desorption/ionization high-resolution mass spectrometry (MALDI-HRMS; Kusari et al., 2015). This study suggested the site of hypericin biosynthesis is in dark nodules and adjacent leaf tissues. The localization of hypericin in dark nodules of the leaves of *Hypericum* spp. cultured *in vitro* was also qualitatively assessed by desorption electrospray ionization mass spectrometry imaging (DESI-MSI). The presence of hypericin in closeness of the dark nodules was confirmed in *H. annulatum*, *H. perforatum*, and *H. tomentosum*, while in *H. androsaemum* and *H. kalmianum* it was not detected (Kucharíková et al., 2016). Hypericin biosynthesis consists of experimentally not yet proven subsequent reactions (**Figure 1**). Acetyl-CoA is condensed with seven molecules of malonyl-CoA to form the octaketide chain. This undergoes specific cyclization to form emodin anthrone, the immediate precursor of hypericin, catalyzed by the octaketide synthase (OKS). Emodin is converted to protohypericin, followed by condensation and transformation reaction leading to hypericin under visible light irradiation (Bais et al., 2003; Zobayed et al., 2006). This study was dedicated to identify new genes involved in the hypericin biosynthesis pathway by approach of functional genomics. Next generation sequencing (NGS) method, especially RNA-Seq (RNA sequencing) used for cDNA identification enables deeper view into biological mechanisms with a potential to reveal unprecedented complexity of the transcriptomes in non-model plants.

To-date, the only available NGS data of the genus *Hypericum* are from *H. perforatum* (St John’s Wort), as the model representative of genus with 39 SRA-NCBI archive entries. The aim of our work was to create new transcriptomic resources for four *Hypericum* spp. (*H. annulatum* and *H. tomentosum* as hypericin-producing and *H. androsaemum* and *H. kalmianum* as hypericin-lacking spp.). Interspecific approach and differential gene expression in leaf tissues with/without dark nodules and hypericin content were performed to approve already identified differentially expressed genes (DEGs) associated with hypericin biosynthesis from *H. perforatum* (Soták et al., 2016). We concentrated especially on verification of the occurrence and expression levels of octaketide synthase *HpPKS2* (OKS; Karppinen et al., 2008) and phenolic oxidative coupling like proteins (POCP) including *hyp-1* sequences (Bais et al., 2003) in leaves. The group of genes coding POCPs belongs to PR-10 genes family (Fernandes et al., 2013). Phenolic oxidative coupling proteins share sequences of SRPBCC (START/RHO_alpha_C/PITP/Bet_v1/CoxG/CalC) domain superfamily.

## Materials and Methods

### Plant Material and RNA Extraction

*Hypericum annulatum* Moris, *H. tomentosum* L., *H. androsaemum* L., and *H. kalmianum* L. plants were cultivated *in vitro* on basal medium containing salts according to Murashige and Skoog (1962), Gamborg’s B5 vitamins (Gamborg et al., 1968), 30 g.l^-1^ sucrose, 100 mg.l^-1^ myoinositol, 2 mg.l^-1^ glycine, and 7 g.l^-1^ agar. The plants were grown in the chamber at 23°C, 40% relative humidity, 16/8 h day/night photoperiod and artificial irradiation of 80 μmol m^-2^ s^-1^. Leaf tissues from 4-week old seedlings were processed on ice under sterile conditions, frozen rapidly in liquid nitrogen and kept at -80°C till the RNA extraction.

**FIGURE 2:**
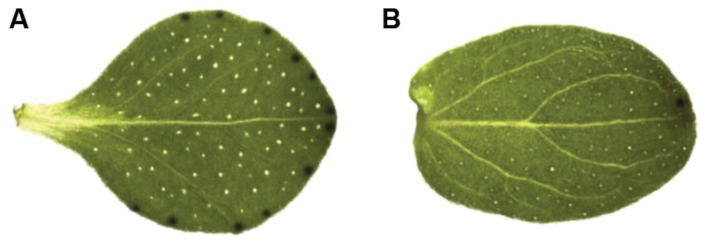
**(A)** *Hypericum annulatum* and **(B)** *H. tomentosum* leaf.

The marginal parts of the leaves with dark nodules (*H. annulatum*) and apical part of leaves with 1–2 dark nodules (*H*. *tomentosum*) were separated from the inner part leaf tissues lacking dark nodules (**Figure 2**). Whole leaves were collected from *H. androsaemum* and *H. kalmianum* seedlings. Each sample contained approximately 10 individual genetically identical plants representing biological replicates from one specimen. The frozen tissues were homogenized by TissueLyser II (Qiagen) and total RNA was extracted with Spectrum^TM^ Plant Total RNA Kit (Sigma–Aldrich) according to manufacturer’s protocol. The quality of total RNA was analyzed on the basis of UV absorption ratios by NanoDrop spectrophotometer 2000 (Thermo Scientific) and the RNA integrity was determined on 2100 Bioanalyzer (Agilent Technologies).

### Determination of Polyketides Content in *Hypericum* spp. Leaves

The experimental design of current transcriptomic paper was based on qualitative metabolomic data of the same *in vitro* cultivated clones by desorption electrospray ionization mass spectrometry imaging (DESI-MSI) published by Kucharíková et al. (2016). The content of phloroglucinol (hyperforin), naphtodianthrones (protopseudohypericin, pseudohypericin, protohypericin, and hypericin) and their putative precursor emodin in the leaves of *Hypericum* species was determined by high-performance liquid chromatography (HPLC). The extracts were prepared and analyzed according to Bruňáková and Čellárová (2016) in three replicates. The compounds were identified and quantified by the Agilent Infinity 1,260 HPLC system (Agilent Technologies, Palo Alto, CA, USA) equipped by a diode array detector. The separation was performed with Agilent Poroshell 120, EC-C18 (3.0 mm × 50 mm, 2.7 μm).

### Illumina Transcriptome Sequencing

Oligo(dT) magnetic beads were used for enrichment of the mRNA from total RNA. The fragmentation of mRNA into short fragments (200 ∼ 500 bp) was conducted by the fragment buffer treatment. The first-strand cDNA was synthesized by random hexamer-primer with the mRNA fragments as templates. Buffer, dNTPs, RNase H, and DNA polymerase I were used to synthesize the second strand. The double strand cDNAs, purified with QiaQuick PCR extraction kit, were used for end repair and base A addition. Short fragments were purified by agarose gel electrophoresis after connecting short fragments to sequencing adapters, and enriched by PCR to create the final cDNA library. The libraries were subjected to RNA-Seq using an Illumina HiSeq^TM^ 2000 platform (BGI Americas, USA). Sequencing was performed in paired-end mode with the read length of 100 bp.

### Quality Control and *De Novo* Assembly Without the Reference Genome

Using the BGI in house software program (BGI Americas, USA), high-quality clean reads were filtered from the raw reads by removing adaptor sequences, low-quality reads with the percentage of unknown nucleotides ‘N’ higher than 5% and reads with the percentage of low quality bases higher than 20% (PHRED quality scores lower than 10). Transcriptome *de novo* assembly was conducted using the program Trinity (v2.0.6; Grabherr et al., 2011) with default settings. The reads were firstly combined based on nucleotide complementarity with certain lengths of overlap to form longer fragments, known as contigs. Contigs redundancy was decreased by the program CD-HIT-EST (Fu et al., 2012), the sequence identity threshold was set to 0.98, word length to 10 and both strands were compared. Sequence Cleaner software (SeqClean) processed and cleaned the contigs at default settings^1^.

### Differential Expression and qRT-PCR Validation

The sequence reads filtered by the quality control were aligned to the assembled contigs using Bowtie2 (Langmead and Salzberg, 2012) to obtain counts of mapped reads. The relative abundances and expected read counts for the contigs were estimated with RNA-Seq by Expectation-Maximization (RSEM; Li and Dewey, 2011). Three different Bioconductor R packages were used to gage important differences in the marginal parts of the leaves with dark nodules and in the inner part leaf tissues without dark nodules of *H. annulatum* and *H*. *tomentosum*: EdgeR (Robinson et al., 2010), DESeq (Anders and Huber, 2010), and NOISeq (Tarazona et al., 2011), a novel non-parametric approach for the identification of DEGs from count data with the ability to simulate replicates. The variance of 0.05 and the *P*-value of 0.05 were set as the thresholds for the significance of the differential gene expression between two types of tissue samples in hypericin producing species.

Five of DEGs upregulated in the leaf rim tissues, namely OKS and four different forms of predicted genes coding POCP were selected to verify differential expression analysis by quantitative real-time polymerase chain reaction (qRT-PCR). The RNA extraction process followed the same protocol as described for RNA-Seq. Degenerative primers were constructed for the reference gene, translation elongation factor 1α (EF-1α; Košuth et al., 2007). The first-strand cDNAs were synthesized from 1 μg of total RNAs using RevertAid Reverse Transcriptase (Thermo Fisher Scientific). NCBI primer-BLAST and Primer3 (Untergasser et al., 2012), a tools for finding specific primers were applied (**Table 1**). LightCycler^®^ Nano Instrument (Roche) was utilized for qRT-PCR reaction and ran on SYBR Green Supermix chemistry. Amplification was performed as follows: first denaturation at 95°C for 10 min, followed by 40 cycles of denaturation at 95°C for 15 s, annealing at 60°C for 10 s and extension at 72°C for 60 s with a single fluorescence measurement. Melting curve program (60–95°C with a heating rate of 0.1°C per second and a continuous fluorescence measurement) and finally a cooling step to 40°C. All the PCR reactions were carried out in three replicates. The relative expression levels for each gene were calculated using the ΔΔC_t_ method.

**Table 1:** Gene-specific primers used for quantitative RT-PCR analyses.

Species	Gene	Contig	Accession number	Primer sequence 5′–3′
*H. annulatum*	*POCP1*	TR34666	KU744672	F CCGATTTCTCCGAGTTTGAA
				R CTCAGGTTTCTCCATCTCCAA
	*POCP2*	TR36949	KU744673	F CCAGTGACCCATTATACCTCAAA
				R CCACACAATACAGCCCTCAA
	*POCP3*	TR41545	KU744674	F CTTGGCTCAAACCCGAAATA
				R GCAAGCCAAAGGTGAAACTC
	*POCP4*	TR45083	—NA—	F GAGGTTTCACTTTCTTCCCTGT
				R CACCCGGCGATTTACACTAC
*H. perforatum*	*POCP1*	TR93881	KU744669	F CCGATTTCTCCGAGTTTGAA
				R CTCAGGTTTCTCCATCTCCAA
	*POCP2*	TR82269	KU744670	F CCAGTGACCCATTATACCACAA
				R GCAACACGATACATCCCTCA
	*POCP3*	TR24220	KU744671	F CTTGGCTCAAACCCGAAATA
				R AAAGGCGAACTCGAACTCAA
	*POCP4*	TR1044	—NA—	F GAGGTTTCACTTTCTTCCCTGT
				R CACCCGGCGATTTACACTAC
*H. tomentosum*	*POCP1*	TR47529	KU744675	F CCGATTTCTCCGAGTTTGAA
				R CACTCAGATTTCTCCATCTCCA
	*POCP2*	TR38948	KU744676	F AACCAGTGACCCATTACACCA
				R GCAACACAATACAGCCCTCA
	*POCP3*	TR8620	KU744677	F CTTGGCTCAAACCCGAAATA
				R GCAAGCCAAAGGTGAAACTC
	*POCP4*	TR5871	—NA—	F TCTCCCCTAACCCACAAAAA
				R GACTTCCACGACACGATTCA


### Functional Annotation and Similarity Search

The transcriptomes of the studied *Hypericum* spp. were annotated against the SwissProt database (Swiss Institute of Bioinformatics databases^2^) and the NCBI non-redundant protein database^3^ using blastx at the *E*-value cut-off of 10^-5^. The results of five best hits were extracted and the hits of lower *E*-value than 10^-5^ were considered to be significant. Functional annotation was performed with the Blast2GO software (Conesa and Götz, 2008). Gene ontology (GO) terms were taxonomically specified to green plants (*Viridiplantae*) and Enzyme Commission (EC) codes were achieved. The annotation was improved by the Augment Annotation by ANNEX function (Myhre et al., 2006), Validate annotation and Remove first level annotation to erase all the redundant GO terms for a given sequence and to assign only the most specific GO terms. Sequence alignments of candidate genes were performed and phylogenetic tree was constructed by the neighbor-joining method with the MEGA 6 program (Tamura et al., 2013).

## Results

### Determination of Polyketides Content by HPLC

High-performance liquid chromatography analysis confirmed the presence of hypericins and emodin in hypericin producing spp., *H. annulatum*, *H. tomentosum*, and *H. perforatum*. The phloroglucinol hyperforin was detected in *H. androsaemum* and *H. perforatum* (**Supplementary Table S1**).

### Sequencing and *De Novo* Assembly

Six cDNA libraries from the marginal parts of the leaves with dark nodules and from the inner part leaf tissue lacking dark nodules from *H. annulatum* and *H*. *tomentosum*, and whole leaves from *H. androsaemum* and *H. kalmianum*, were subjected to Illumina sequencing. Paired-end read technology was preferred to increase the depth and improve *de novo* assembly efficiency. Sequencing using the Illumina HiSeq^TM^ 2000 platform resulted in the generation of 74.4 G raw reads. The samples were deposited in NCBI Sequence Read Archive (SRA) with accession numbers SRX1528960 (*H. annulatum* leaves with dark nodules), SRX1528962 (*H. annulatum* leaves without dark nodules), SRX1528963 (*H. tomentosum* leaves with dark nodules), SRX1528964 (*H. tomentosum* leaves without dark nodules), SRX1528157 (*H. androsaemum*) and SRX1528958 (*H. kalmianum*). After removing reads with adapters, reads with unknown nucleotides, and low-quality reads, we gained 312.4 million clean reads with the average GC content of 50.56% and more than 98% of the bases had PHRED quality scores higher than Q20 (error rate < 0.01%; **Table 2**).

**Table 2:** Statistics of the sequencing output and preprocessed data.

Sample description		Read quality							
	
*Hypericum* species	Material	Total reads (Gb)	%Q20 before filter	%Q20 after filter	%GC before filter	%GC after filter	Filter adapter	Filter low quality	Clean reads (Gb)
*H. annulatum*	Marginal parts of the leaves with dark nodules	5.75	96.88	98.25	50.23	50.21	3.24	2.79	5.4
*H. annulatum*	Inner part of leaves without dark nodules	5.83	96.63	98.15	50.93	50.87	3.2	2.97	5.47
*H. tomentosum*	Apical parts of the leaves with dark nodules	4.56	96.58	98.24	50.18	50.13	4.04	2.91	4.24
*H. tomentosum*	Inner parts of the leaves without dark nodules	6.42	96.67	98.16	50.35	50.3	3.88	3.05	5.98
*H. androsaemum*	Whole leaves	5.6	96.76	98.16	54.49	51.45	3.19	2.94	5.26
*H. kalmianum*	Whole leaves	5.21	96.94	98.27	50.46	50.42	3.49	2.71	4.89


The clean reads were assembled by the Trinity program to obtain the *de novo* reference transcriptome sequence. The first leaf transcriptome of *H. androsaemum*, *H. annulatum*, *H. kalmianum*, and *H. tomentosum* was generated. The CD-HIT program followed by SeqClean removed redundant and similar isoforms. The high-quality transcriptomes of different *Hypericum* ssp. were created and described in **Table 3**.

**Table 3:** Statistics of transcriptome *de novo* assembly.

*Hypericum* species	Total assembled bases	Total trinity genes	Contig N50	Average contig	Percent GC (%)
*H. annulatum*	91,602,062	60,611	1771	1077.27	45.04
*H. tomentosum*	92,533,952	59,872	1851	1131.87	44.89
*H. androsaemum*	84,822,534	60,041	1602	991.01	46.08
*H. kalmianum*	74,609,783	51,244	1642	1018.51	45.19


### Identification and Validation of Differentially Expressed Contigs

**FIGURE 3:**
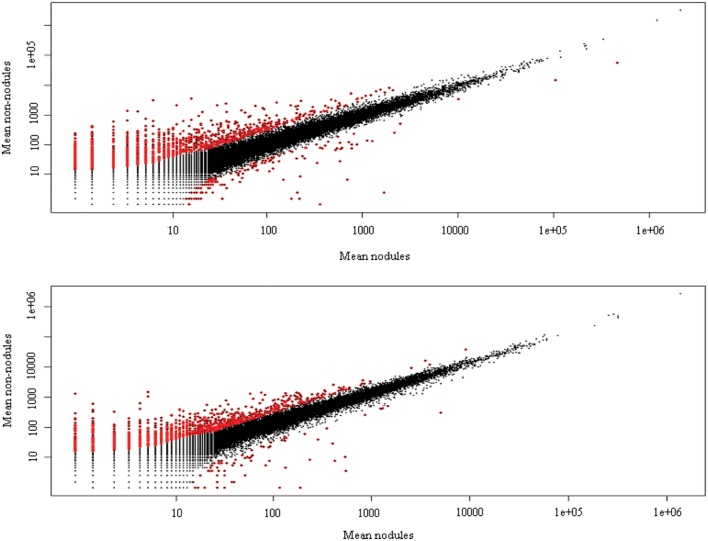
**Mean plot of DEGs analysis between marginal parts of leaves with dark nodules and inner part of leaves without nodules of *H. annulatum* and *H. tomentosum*.** Red dots show significantly different gene expression and black dots indicate no significant differences. Nodules: dark nodules with adjacent leaf tissue, non-nodules: leaf tissue lacking dark nodules.

**FIGURE 4:**
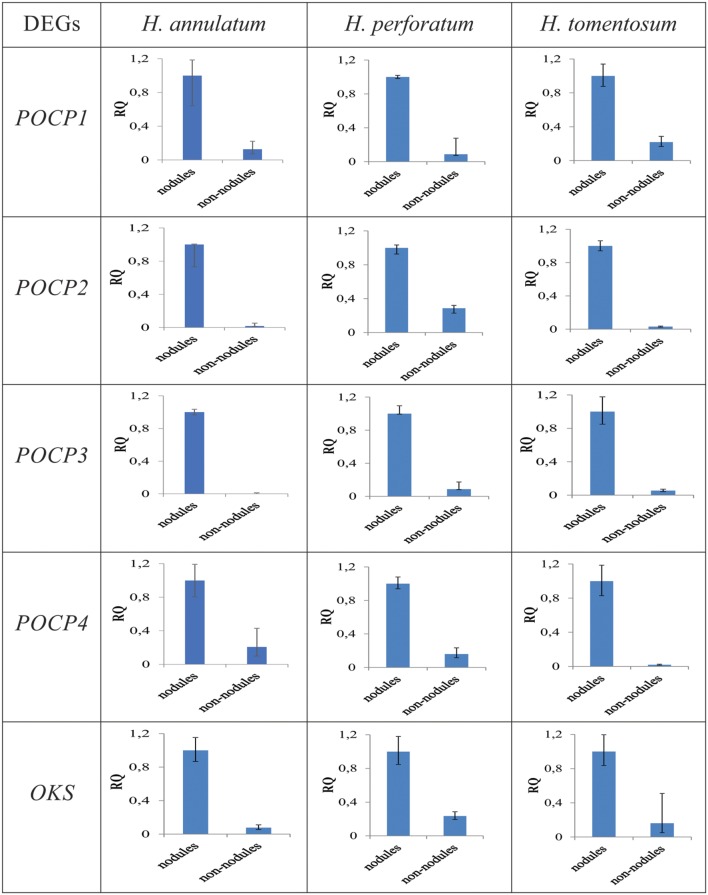
**Quantitative real-time polymerase chain reaction (qRT-PCR) validation of expression levels of genes coding POCPs and OKSs.** Nodules: dark nodules with adjacent leaf tissue, non-nodules: leaf tissue lacking dark nodules. RQ = relative quantification.

Cleaned reads of *H. annulatum* and *H. tomentosum* as the hypericin producers were separately aligned (Bowtie2) to *de novo* assembled transcriptomes. Transcripts abundances were normalized using RSEM package and fragments per kilobase of transcript per million fragments mapped value (FPKM) were estimated. Differential expression analyses between the parts of the leaves with dark nodules and the inner part leaf tissues without dark nodules were performed for both species separately. DE analysis ran on the basis of three different R packages (DESeq, edgeR, and NOISeq) at the values of var = 0.05 and *P* < 0.05 (**Figure 3**). We attained 165 contigs (135 DEGs) for *H. annulatum* and 100 contigs (72 DEGs) for *H. tomentosum* upregulated in the leaf tissues containing dark nodules (**Supplementary Tables S2** and **S3**). The qRT-PCR was applied for experimental confirmation of the computational analysis. Five DEGs with significant transcript abundance changes were subjected to the validation. Normalized relative ratios based on ΔΔC_t_ method were calculated to estimate relative quantification of the expression level. The expression patterns in qRT-PCR were in agreement with the transcriptomic data for all the detected genes (**Figure 4**).

### Functional Annotation and Classification

**FIGURE 5:**
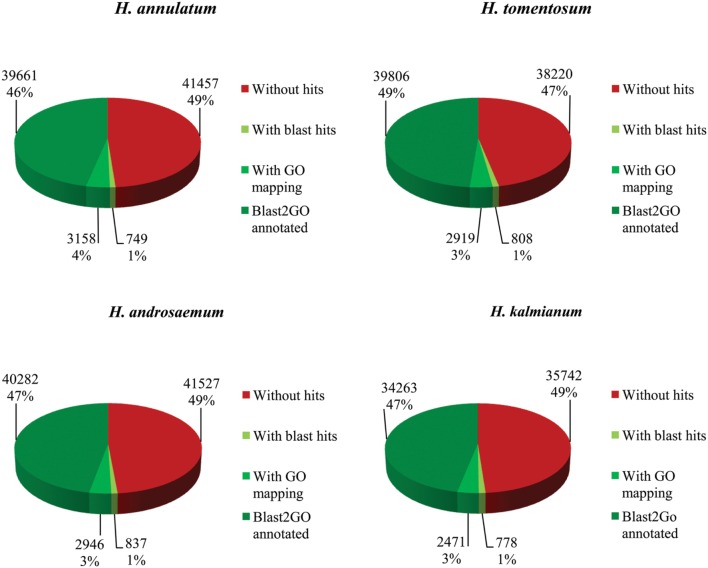
**Blast2GO annotation results for *Hypericum* spp. against SwissProt database**.

Gene categorization was conducted by homology search blastx against SwissProt and NCBI non-redundant protein database (NCBI-nr) with a cut-off *E*-value of 1 × 10^-5^. SwissProt database was used to process basic functional information for the transcriptomes from all four species. Due to limited genomic information about the selected *Hypericum* spp. only lower coverage of annotated data was achieved. Approximately 50% of contigs showed blastx hit to SwissProt (**Figure 5**).

**FIGURE 6:**
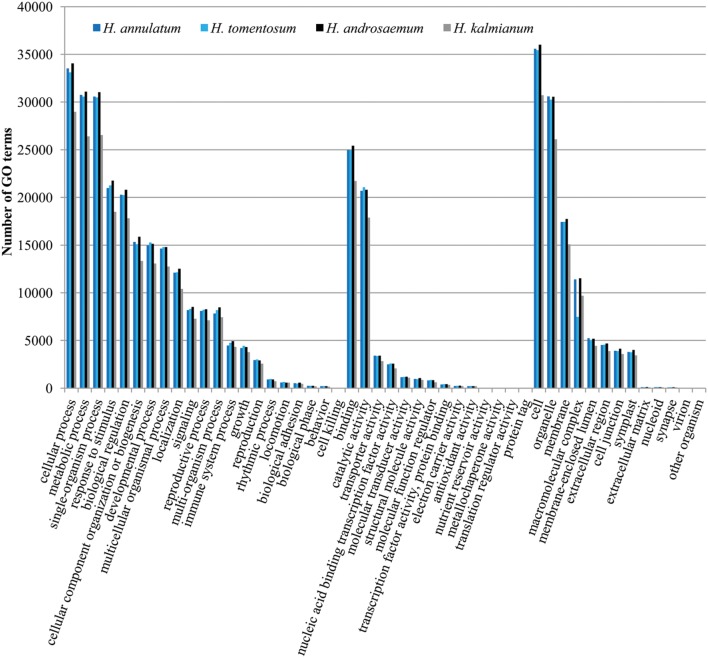
**Gene ontology (GO) classification comparison of the four analyzed *Hypericum* spp. of SwissProt annotated contigs**.

We presume the key enzymes for most of the processes including secondary metabolism of specific compounds share similar coding sequences. GO classification was carried out to show cellular component, molecular function and biological process primary distribution (**Figure 6**). Highly similar numbers of GO terms for all four studied *Hypericum* spp. were discovered. The most frequent subcategories in Biological Process section were cellular, metabolic and single organism processes. The most common terms in Molecular Function were binding and catalytic activities.

**FIGURE 7:**
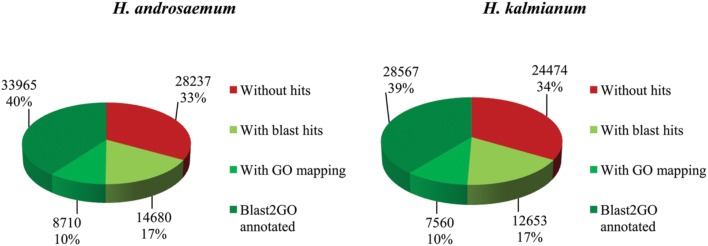
***Hypericum androsaemum* and *H. kalmianum* annotation statistics of blastx against NCBI-nr database**.

**FIGURE 8:**
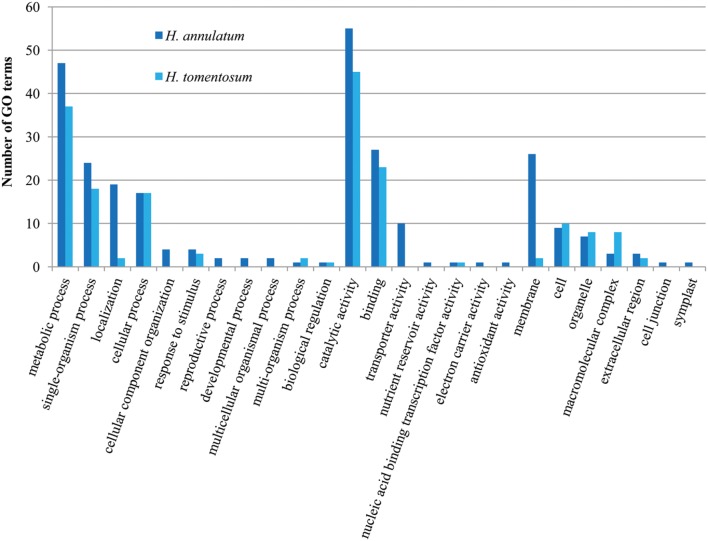
**Gene ontology classification of NCBI-nr annotated *H. annulatum* (135 DEGs) and *H. tomentosum* (72 DEGs)**.

The transcriptomes of *H. androsaemum* and *H. kalmianum* were annotated also to NCBI-nr and used as the negative control to contigs originated from hypericin producing *H. annulatum* and *H. tomentosum* DEGs. Almost 70% of contigs showed blastx hit to NCBI-nr (**Figure 7**). Hypericin producing species *H. annulatum* and *H. tomentosum* DEGs upregulated in leaf rim containing dark nodules were blasted against NCBI-nr additionally. Functional annotation was improved as the SwissProt database did not cover rich variety of not yet verified genes from non-model species. The most frequent GO subcategories for DEGs were metabolic process and catalytic activity. The distribution of the major GO subcategories in all of the *Hypericum* spp. for NCBI-nr annotated transcriptomes was comparable (**Figure 8**).

**FIGURE 9:**
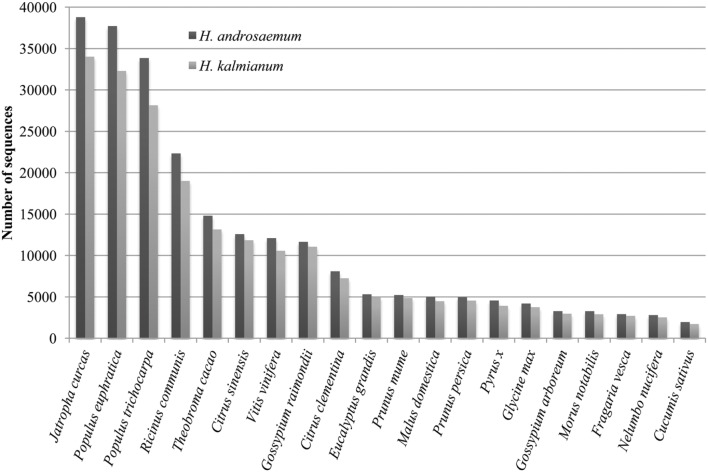
**Species distribution**.

Blast2GO analysis of species distribution showed unexpected transcriptome similarity of *Hypericum* spp. to *Jatropha curcas* L. despite of considerable taxonomical distance. *Hypericum* spp. are known for its similarity to genera *Populus*, *Ricinus*, and *Vitis vinifera* of which rRNAs sequences are used to mRNA set clean up (**Figure 9**).

### Contigs Supposedly Related to Hypericin Biosynthesis

The genetic and metabolomic background of hypericin biosynthesis led us to lay our interest in two groups of crucial enzymes, OKSs and POCPs. *HpPKS2*, the gene coding OKS was expressed in dark nodules (Karppinen et al., 2008). OKSs are supposed to play key role in the reaction of emodin anthrone formation. The gene coding POCP (*hyp-1*) has been originally reckoned as the gene capable to catalyze conversion of precursor emodin to hypericin (Bais et al., 2003). Although this presumption has never been proved, it is possible, the different gene variant coding POCP homologous to *hyp-1* may catalyze this reaction.

The sequence alignment of the *HpPKS2* (EU635882.1) to transcriptomes discovered presence of five contigs in *H. perforatum* (Soták et al., 2016), six contigs in *H. annulatum*, two contigs in *H. tomentosum*, three contigs in *H. androsaemum* and one single contig in *H. kalmianum*. The sequence homology search identified DEGs annotated as genes coding OKS in *H. annulatum* (3 contigs), *H. perforatum* (1 contig), and *H. tomentosum* (2 contigs). The sequence alignment of hypericin producing *Hypericum* spp. discovered one particular OKS DEG with only little variation and meaningful match to *HpPKS2* published by Karppinen et al. (2008) with sequence identity higher than 97%. *H. kalmianum* and *H. androsaemum* shared one similar gene coding OKS with sequence different from OKS genes found in hypericin producing species and Karppinen’s *HpPKS2* with the sequence identity about 70%.

**FIGURE 10:**
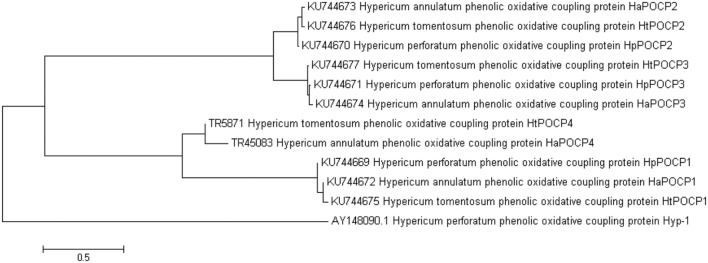
**Neighbor joining tree of POCP sequences**.

This transcriptomic study demonstrated the existence of more genes and isoforms resembling to POCP *hyp-1* sequence (AY148090.1). Blast2GO annotation of leaf transcriptomes against NCBI-nr database revealed genes supposedly coding POCPs in both hypericin producing and non-producing species: *H. annulatum* (8 genes, 9 isoforms), *H. perforatum* (14 genes, 21 isoforms) and *H. tomentosum* (15 genes, 17 isoforms), *H. androsaemum* (14 genes, 20 isoforms), *H. kalmianum* (8 genes, 16 isoforms), highly similar to *hyp-1*. The sequence homology analysis of DEGs in hypericin producing species detected three different gene forms supposedly coding POCPs common for *H. perforatum* (KU744669, KU744670, and KU744671), *H. annulatum* (KU744672, KU744673, and KU744674) and *H. tomentosum* (KU744675, KU744676, and KU744677) and one form common for *H. annulatum* and *H. tomentosum* (**Figure 10**). NCBI Open Reading Frame Finder identified the coding sequences of the same length in predicted POCP gene forms: POCP1 (471 bp), POCP2 (483 bp), POCP3 (477 bp), and POCP4 (480 bp). These lengths corresponded to the length of *hyp-1* coding sequence from *H. perforatum* (480 bp). The sequence identity within each proposed gene coding POCP varied from 95 to 98% and showed significant divergence to *hyp-1* sequence. *Hyp-1* did not occur among DEGs upregulated in tissues containing dark nodules.

## Discussion

The presented paper contributes to fill the blank space in transcriptome profiling of *Hypericum* spp. Nowadays, the only transcriptomic information within the genus *Hypericum* is available for *H. perforatum*, representing the “model” species of the genus. This is the first high throughput RNA-Seq description of *H. annulatum, H. tomentosum, H. androsaemum*, and *H. kalmianum* transcriptomes. Our major interest was aimed at identification of secondary metabolites coding genes especially those associated to naphtodianthrone hypericin. Differential expression analysis on RNA-Seq data was selected to uncover contigs upregulated in the leaf parts containing dark nodules and adjacent tissues. The experimental selection of contrasting leaf tissues with/without dark nodules (leaf rim vs. leaf middle) for differential gene expression analysis was based on the latest metabolomic results. Kusari et al. (2015) analyzed leaves excised from *ex vitro* cultivated *H. perforatum* plants by MALDI-HRMS method. Kucharíková et al. (2016) used DESI-MSI for qualitative characterization of metabolome of the leaf tissues from 17 *Hypericum* spp. including same *in vitro* cultured clones as in the current transcriptomic study. The occurrence of hypericin in proximity of dark nodules was approved in *H. annulatum*, *H. perforatum*, and *H. tomentosum*, while in *H. androsaemum* and *H. kalmianum* it was not detected. According to metabolomic data it is predicted that at least the final steps of hypericin biosynthesis are situated to the leaf parts containing nodules and adjacent tissues. HPLC analysis added quantitative values of polyketides content of selected *Hypericum* spp. leaves.

Three different Bioconductor packages listed 165 contigs in *H. annulatum* and 100 contigs in *H. tomentosum* as significantly differentially expressed (*P* < 0.05) and upregulated in the leaf rim tissues containing the dark nodules. The number of DEGs upregulated in leaf tissues containing dark nodules was significantly lower in comparison to DESeq2 analysis of two technical replicates *H. perforatum* revealing 799 DEGs sequences with 2058 isoforms (Soták et al., 2016). As the current analysis ran solely on the biological replicates, NOISeq-sim script from the NOISeq package simulated technical replicates in this interspecific approach survey and the final results showed only a smaller number of highly significant DEGs hits.

According to the GO classification of the contigs, the section Biological Process, subcategory Metabolic Process represented the second largest group for all four *Hypericum* spp. and for DEGs upregulated in leaf tissues containing dark nodules appeared as the most frequent group. These classification findings highly correspond to the recent studies on *H. perforatum* transcriptome from the whole plant (He et al., 2012) and the transcriptome from leaf tissues (Soták et al., 2016). On the other hand, a comparison between hyperforin-producing *H. annulatum* and hyperforin-lacking *H. tomentosum* revealed a significant difference in the section Macromolecular complex.

The sequence analysis of DEGs in *H. perforatum*, *H. annulatum*, *H. tomentosum*, and hypericin non-producing *H. kalmianum* and *H. androsaemum* (NCBI-nr annotated) including already published sequences in NCBI-nr database showed meaningful inter and intra species variations for both of focused enzymes. We investigated the presence and expression levels of the *HpPKS2* (Karppinen et al., 2008) and *hyp-1* (Bais et al., 2003). Contigs coding OKSs were found in all *Hypericum* transcriptomes but OKS gene sequences from hypericin non-producing species were more different from *HpPKS2*. We suppose that OKS gene sequences identified among DEGs in hypericin producing species may play a role in hypericin biosynthesis. Differential gene expression analysis revealed four novel genes supposedly coding POCP belonging to PR-10 class proteins. Although four different gene forms of the proposed POCPs were annotated in DEGs, none of them was highly similar to *hyp-1*. The whole transcriptome annotation of *H. perforatum* against NCBI-nr discovered 14 genes, 21 isoforms coding PR-10 proteins. Karppinen et al. (2016) identified four different genes coding PR-10: *HpPR10.2*, *HpPR10.3*, *HpPR10.4*, and *HpPR10.1* representing *hyp-1* sequence. The transcriptomic study of *H. perforatum* validated the presence of all sequences published by Karppinen et al. (2016).

The *hyp-1* was also expressed in the other 15 *Hypericum* species regardless of whether hypericins and emodin were detected in the plants (Košuth et al., 2011). We assume, though the *hyp-1* was not found in DEGs of hypericin producing species but was present only in the transcriptome datasets of all four studied *Hypericum* spp. regardless they synthesize hypericin or not, the probability of *hyp-1* to represent the crucial component of emodin to hypericin conversion is doubtful.

## Conclusion

This study was objected to elucidate hypericin biosynthesis pathway by approach of interspecific differential gene expression analysis of leaf tissues with/without dark nodules and hypericin-producing and hypericin-lacking species. We have generated six new RNA-Seq libraries of *Hypericum* spp. followed by sequencing and bioinformatic analysis with the focus on biological properties to reveal unknown features of genes, proteins, metabolites, and biological pathways. The results proved existence of the most of differentially expressed contigs in *H. annulatum*, *H. perforatum*, and *H. tomentosum*, as they supposedly share the same hypericin biosynthetic pathway steps. Sequence alignment homology of DEGs upregulated in tissues containing dark nodules identified presence of contigs highly similar to *HpPKS2*. Hypericin non-producing species uncovered contigs with only low homology to *HpPKS2*. New variants of cDNAs coding enzyme catalyzing phenolic oxidative coupling reactions were recognized in the dataset of DEGs from the tested hypericin producing species. We suppose that different variant of proposed POCP may play a role in hypericin biosynthesis. This presumption will be further verified by functional validation experimental approaches.

## Availability of Supporting Data

The RNA sequence dataset supporting the results in this article are available in the NCBI Sequence Read Archive. The accession number are SRX1528960 (*H. annulatum* leaves with dark nodules), SRX1528962 (*H. annulatum* leaves without dark nodules), SRX1528963 (*H. tomentosum* leaves with dark nodules), SRX1528964 (*H. tomentosum* leaves without dark nodules), SRX1528157 (*H. androsaemum*) and SRX1528958 (*H. kalmianum*). The nucleotide sequence of genes coding POCPs are available in the NCBI GenBank submitted through Banklt for *H. annulatum* (KU744672, KU744673, and KU744674), *H. perforatum* (KU744669, KU744670, and KU744671) and *H. tomentosum* (KU744675, KU744676, and KU744677).

## Author Contributions

MS designed the study, concluded the analyses and drafted the manuscript. OC prepared samples, analyzed the data, performed the validations, and drafted the manuscript. DK conducted the DEG analysis. ZJ analyzed the data and performed the validations. LL participated on Illumina sequencing, revised the manuscript. EC designed the study, directed on the study and revised the manuscript. All authors read and approved the final manuscript.

## Conflict of Interest Statement

The authors declare that the research was conducted in the absence of any commercial or financial relationships that could be construed as a potential conflict of interest.

The handling Editor declared an ongoing collaboration, the co-editing of a Frontiers Research Topic, with one of the authors EC, but states that no other conflict of interest exists and that the review process was nevertheless fair and objective.

## Footnotes

1 https://sourceforge.net/projects/seqclean

2 http://www.uniprot.org/downloads

3 ftp://ftp.ncbi.nlm.nih.gov/blast/db/

## Supplementary Material

**TABLE S1 | Polyketides content measured by HPLC (μg.mg^-1^).**

**TABLE S2 | Counts and FPKM values of *H. annulatum* DEGs annotated on NCBI-nr.**

**TABLE S3 | Counts and FPKM values of *H. tomentosum* DEGs annotated on NCBI-nr.**

## Abbreviations

DEGs: differentially expressed genes
HpPKS2: *Hypericum perforatum* polyketide synthase 2
OKS: octaketide synthase
POCP: phenolic oxidative coupling protein
PR-10: pathogenesis-related class 10
