# Supplementary material for: Comparative Transcriptome Reconstruction of Four Hypericum Species Focused on Hypericin Biosynthesis
Source: Front Plant Sci. 2016 Jul 13;7:1039. doi: 10.3389/fpls.2016.01039 (PMC4942478; doi:10.3389/fpls.2016.01039)
Supplement: Supplementary file 1 [file Table_1.DOCX]

| *Hypericum* species | hypericins±SD | emodin±SD | hyperforin±SD |
| --- | --- | --- | --- |
| *H. annulatum* | 2.06±0.11 | 0.04±0.02 | ND |
| *H. perforatum* | 5.41±0.14 | 0.40±0.01 | 16.69±3.41 |
| *H. tomentosum* | 0.27±0.01 | 0.02±0.01 | ND |
| *H. androsaemum* | ND | ND | 12.35±1.51 |
| *H. kalmianum* | ND | ND | ND |

ND: not detected, SD: standard deviation
