# Supplementary material for: Comparative Transcriptome Reconstruction of Four Hypericum Species Focused on Hypericin Biosynthesis
Source: Front Plant Sci. 2016 Jul 13;7:1039. doi: 10.3389/fpls.2016.01039 (PMC4942478; doi:10.3389/fpls.2016.01039)
Supplement: Supplementary file 2 [file Table_2.DOCX]

| ***H. annulatum* contig** | **NCBI-nr annotation** | **non-nodules FPKM** | **nodules FPKM** | **non-nodules counts** | **nodules counts** |
| --- | --- | --- | --- | --- | --- |
| TR21386\|c0_g1 | ---NA--- | 0,04 | 36,877 | 2 | 1688 |
| TR134\|c1_g1 | membrane protein | 0,41 | 48,942 | 6 | 704 |
| TR41666\|c0_g1 | conserved protein | 0 | 42,505 | 0 | 362 |
| TR11703\|c0_g1 | orf16-lacz fusion partial | 0,21 | 46,763 | 1 | 219 |
| TR34144\|c0_g1 | hypothetical protein WCM_04769 | 0,32 | 33,958 | 2 | 210 |
| TR5609\|c0_g1 | cell wall-associated hydrolase domain partial | 0,23 | 41,685 | 1 | 182 |
| TR44249\|c0_g1 | beta-glucosidase 17-like isoform x6 | 0,71 | 15,794 | 27 | 589 |
| TR32265\|c0_g1 | senescence-specific cysteine protease sag39-like | 0,29 | 8,357 | 8 | 226 |
| TR17169\|c0_g2 | ---NA--- | 0,84 | 20,912 | 6 | 148 |
| TR37352\|c0_g1 | pollen ole e 1 allergen and extensin family | 1,691 | 24,161 | 24 | 338 |
| TR32114\|c0_g1 | polyketide synthase | 10,324 | 107,721 | 75 | 773 |
| TR36949\|c0_g1 | phenolic oxidative coupling protein | 8,023 | 79,251 | 131 | 1278 |
| TR7618\|c0_g1 | polyketide synthase | 9,343 | 83,03 | 246 | 2159 |
| TR12536\|c0_g1 | ---NA--- | 1313,857 | 11171,896 | 55354 | 464733 |
| TR8889\|c0_g1 | s-adenosyl-l-methionine-dependent methyltransferases superfamily | 3,491 | 31,079 | 100 | 880 |
| TR45083\|c0_g1 | major allergen pru ar 1-like | 7,133 | 58,699 | 138 | 1124 |
| TR47071\|c0_g2 | glutathione s-transferase u17-like | 4,142 | 34,477 | 72 | 592 |
| TR16912\|c0_g1 | ---NA--- | 183,186 | 1350 | 14410 | 104851 |
| TR53346\|c0_g1 | ---NA--- | 277,431 | 1894,662 | 14766 | 105446 |
| TR22980\|c0_g1 | 2-oxoglutarate and fe -dependent oxygenase superfamily isoform 1 | 3,461 | 27,1 | 100 | 772 |
| TR34666\|c0_g1 | phenolic oxidative coupling protein | 2,671 | 21,782 | 65 | 523 |
| TR41545\|c0_g2 | phenolic oxidative coupling protein | 4,392 | 34,997 | 78 | 614 |
| TR16880\|c0_g1 | major allergen pru ar 1 isoform 1 | 4,041 | 29,939 | 75 | 549 |
| TR46960\|c0_g1 | polyketide synthase | 7,373 | 54,37 | 34 | 248 |
| TR6274\|c0_g1 | dihydrofolate reductase-like protein | 5,312 | 32,368 | 121 | 728 |
| TR52511\|c0_g3 | truncated transcription factor cauliflower a-like | 0,64 | 5,768 | 12 | 106 |
| TR14355\|c0_g1 | sugar transporter erd6-like 5 | 0,62 | 5,708 | 15 | 116 |
| TR8770\|c0_g1 | glycosyl hydrolase family 1 family protein | 0,78 | 4,888 | 33 | 204 |
| TR15766\|c0_g2 | homolog subfamily c member 2 | 30,981 | 158,343 | 497 | 2512 |
| TR19899\|c0_g1 | thaumatin-like protein | 1,721 | 10,276 | 40 | 235 |
| TR24768\|c0_g1 | 3-glucanase family protein | 4,292 | 22,422 | 114 | 588 |
| TR47657\|c0_g1 | ran bp2 nzf zinc finger-like superfamily protein | 5,402 | 29,789 | 73 | 385 |
| TR25709\|c0_g1 | isoform 1 | 0,04 | 1,459 | 1 | 34 |
| TR29451\|c0_g1 | floral homeotic protein deficiens isoform 1 | 0,26 | 2,769 | 5 | 52 |
| TR47657\|c0_g2 | ran bp2 nzf zinc finger-like superfamily protein | 3,611 | 17,174 | 77 | 361 |
| TR51822\|c0_g1 | gdt1-like protein 5 | 9,974 | 44,834 | 93 | 413 |
| TR6670\|c0_g1 | blue copper | 0,1 | 1,23 | 3 | 38 |
| TR19099\|c0_g2 | ---NA--- | 0,85 | 6,758 | 7 | 55 |
| TR24968\|c0_g1 | fatty acyl- reductase 2-like | 0,65 | 3,979 | 13 | 80 |
| TR57331\|c0_g1 | fatty acyl- reductase 2-like isoform x2 | 0,58 | 3,569 | 13 | 79 |
| TR12968\|c0_g1 | ugt protein isoform 1 | 0,72 | 3,569 | 27 | 133 |
| TR49980\|c0_g1 | ---NA--- | 2,021 | 10,706 | 25 | 116 |
| TR25939\|c0_g1 | pleiotropic drug resistance protein 3 | 0,19 | 1,419 | 6 | 44 |
| TR34518\|c0_g1 | glutathione s- | 0,22 | 2,029 | 4 | 36 |
| TR39937\|c0_g1 | glutathione s-transferase l3-like isoform x1 | 10,074 | 37,286 | 225 | 822 |
| TR12760\|c0_g1 | hypothetical protein POPTR_0014s16050g | 0,21 | 2,839 | 2 | 27 |
| TR44573\|c0_g1 | ---NA--- | 0,63 | 5,428 | 4 | 34 |
| TR10633\|c0_g3 | PREDICTED: uncharacterized protein LOC102618481 isoform X1 | 0,59 | 3,359 | 10 | 56 |
| TR30948\|c0_g2 | atp-binding cassette | 1,25 | 5,058 | 126 | 443 |
| TR50234\|c0_g1 | ---NA--- | 0,63 | 5,298 | 4 | 33 |
| TR48495\|c0_g1 | calmodulin binding | 0,4 | 2,259 | 9 | 50 |
| TR37874\|c0_g1 | 2-oxoglutarate and fe -dependent oxygenase superfamily isoform 1 | 0,12 | 2,439 | 1 | 20 |
| TR21577\|c0_g1 | ---NA--- | 0 | 0,86 | 0 | 15 |
| TR50519\|c0_g1 | low temperature-induced protein | 2,161 | 9,696 | 16 | 71 |
| TR53269\|c0_g1 | ---NA--- | 5,522 | 21,942 | 27 | 106 |
| TR42496\|c0_g1 | glutathione s-transferase | 5,362 | 18,233 | 98 | 329 |
| TR36511\|c0_g1 | ---NA--- | 5,432 | 21,992 | 24 | 96 |
| TR22980\|c0_g2 | 2-oxoglutarate and fe -dependent oxygenase superfamily isoform 1 | 0,77 | 3,569 | 14 | 64 |
| TR4102\|c0_g1 | glutathione s-transferase u17-like | 3,701 | 17,124 | 14 | 64 |
| TR15673\|c0_g1 | ---NA--- | 0,11 | 2,069 | 1 | 19 |
| TR30011\|c0_g2 | purple acid phosphatase 18 | 1,13 | 13,055 | 2 | 23 |
| TR6311\|c0_g1 | pathogenesis-related protein 1-like | 0,15 | 1,749 | 2 | 23 |
| TR12896\|c0_g2 | flavone synthase | 0,46 | 1,949 | 18 | 76 |
| TR15569\|c0_g1 | ---NA--- | 5,342 | 18,193 | 77 | 259 |
| TR19363\|c0_g1 | zeaxanthin chloroplastic-like | 1,651 | 6,328 | 29 | 110 |
| TR11614\|c0_g1 | nadh dehydrogenase subunit 5 | 16,086 | 50,082 | 3267 | 10101 |
| TR10125\|c0_g1 | ---NA--- | 1,2 | 6,018 | 10 | 50 |
| TR26932\|c0_g1 | glutathione s-transferase u17-like | 3,891 | 17,844 | 13 | 59 |
| TR47071\|c0_g1 | glutathione s-transferase u17-like | 4,222 | 13,975 | 78 | 255 |
| TR41582\|c0_g1 | ---NA--- | 0,18 | 3,259 | 1 | 18 |
| TR38183\|c0_g2 | ---NA--- | 0,32 | 2,689 | 3 | 25 |
| TR4984\|c0_g1 | ---NA--- | 0,65 | 2,629 | 18 | 72 |
| TR23521\|c0_g1 | ---NA--- | 3,031 | 11,376 | 24 | 89 |
| TR46769\|c0_g1 | polygalacturonase at1g48100-like | 0,54 | 2,109 | 20 | 77 |
| TR11526\|c0_g1 | gdsl esterase lipase apg | 1,1 | 3,879 | 33 | 115 |
| TR12418\|c0_g1 | ---NA--- | 0,59 | 6,258 | 2 | 21 |
| TR11541\|c0_g1 | heparanase-like protein 3 isoform x1 | 4,222 | 13,355 | 183 | 553 |
| TR53627\|c0_g1 | ---NA--- | 3,191 | 10,796 | 41 | 137 |
| TR23183\|c0_g2 | sugar transporter erd6-like 5-like isoform x1 | 0,12 | 2,109 | 1 | 17 |
| TR31284\|c1_g3 | transmembrane ascorbate ferrireductase 1-like | 1,02 | 4,468 | 12 | 52 |
| TR49464\|c0_g1 | ---NA--- | 0,23 | 1,889 | 3 | 24 |
| TR31613\|c0_g1 | glutathione s-transferase u17-like | 3,951 | 13,995 | 28 | 98 |
| TR23316\|c0_g1 | ---NA--- | 0,25 | 1,809 | 4 | 27 |
| TR3855\|c0_g4 | cytochrome c oxidase subunit 2 | 2,261 | 7,267 | 58 | 184 |
| TR33986\|c0_g1 | ---NA--- | 0,2 | 1,19 | 5 | 30 |
| TR10960\|c0_g1 | rhicadhesin receptor family protein | 1,641 | 7,267 | 11 | 48 |
| TR4995\|c0_g1 | ---NA--- | 0,14 | 1,469 | 2 | 20 |
| TR11050\|c0_g1 | phosphoethanolamine phosphocholine | 2,361 | 7,637 | 42 | 134 |
| TR14348\|c0_g2 | ---NA--- | 0,06 | 0,97 | 1 | 16 |
| TR33680\|c0_g1 | ---NA--- | 0,08 | 1,29 | 1 | 16 |
| TR951\|c0_g1 | ---NA--- | 0,49 | 3,219 | 4 | 26 |
| TR37058\|c0_g1 | ---NA--- | 0,47 | 2,789 | 5 | 29 |
| TR39964\|c0_g1 | ---NA--- | 1,871 | 6,088 | 37 | 119 |
| TR113\|c0_g1 | rhamnogalacturonate lyase family protein | 0,6 | 2,179 | 19 | 68 |
| TR10633\|c0_g2 | PREDICTED: uncharacterized protein LOC102618481 isoform X1 | 1,17 | 4,129 | 21 | 73 |
| TR20334\|c0_g2 | auxin-responsive protein iaa29-like | 1,751 | 5,278 | 60 | 179 |
| TR12588\|c0_g1 | ---NA--- | 2,461 | 7,307 | 50 | 151 |
| TR270\|c0_g1 | ---NA--- | 1,16 | 3,809 | 28 | 91 |
| TR22575\|c2_g3 | ---NA--- | 4,712 | 19,913 | 10 | 42 |
| TR19350\|c0_g1 | ---NA--- | 0,07 | 1,13 | 1 | 15 |
| TR20145\|c0_g2 | ---NA--- | 0,14 | 2,089 | 1 | 15 |
| TR39693\|c0_g2 | phosphoethanolamine phosphocholine | 10,914 | 33,688 | 37 | 113 |
| TR31159\|c0_g1 | ---NA--- | 0,22 | 1,989 | 2 | 18 |
| TR23375\|c0_g1 | aldo keto | 2,331 | 7,297 | 34 | 105 |
| TR13404\|c0_g1 | reticuline oxidase | 0,33 | 1,829 | 5 | 27 |
| TR39691\|c0_g1 | ---NA--- | 1,33 | 7,267 | 5 | 27 |
| TR43957\|c0_g2 | ---NA--- | 0,24 | 1,459 | 4 | 24 |
| TR24462\|c0_g2 | ---NA--- | 3,471 | 10,636 | 34 | 103 |
| TR50640\|c0_g1 | sorbin and sh3 domain-containing protein 2-like | 0,28 | 1,35 | 6 | 29 |
| TR27795\|c0_g1 | ---NA--- | 1,871 | 9,806 | 5 | 26 |
| TR1476\|c0_g1 | gamma-secretase subunit aph1-like | 0,38 | 2,209 | 4 | 23 |
| TR962\|c0_g1 | hypothetical protein POPTR_0005s08920g | 1,22 | 7,107 | 4 | 23 |
| TR25110\|c0_g1 | ---NA--- | 0,21 | 1,779 | 2 | 17 |
| TR38945\|c0_g1 | ---NA--- | 0,51 | 4,378 | 2 | 17 |
| TR49136\|c0_g1 | ---NA--- | 0,2 | 1,709 | 2 | 17 |
| TR36523\|c0_g1 | probable disease resistance protein at4g27220 | 0,78 | 5,288 | 3 | 20 |
| TR38201\|c0_g1 | ---NA--- | 0,76 | 5,148 | 3 | 20 |
| TR49376\|c0_g1 | ---NA--- | 0,5 | 2,369 | 6 | 28 |
| TR51280\|c0_g1 | ---NA--- | 0,6 | 2,829 | 6 | 28 |
| TR8454\|c0_g1 | exocyst complex component sec8-like | 1,15 | 5,828 | 5 | 25 |
| TR39693\|c0_g1 | phosphoethanolamine phosphocholine | 1,401 | 5,848 | 8 | 33 |
| TR56587\|c0_g1 | ---NA--- | 1,561 | 8,677 | 4 | 22 |
| TR46539\|c0_g1 | ---NA--- | 1,901 | 12,076 | 3 | 19 |
| TR14678\|c0_g1 | ---NA--- | 0,49 | 3,949 | 2 | 16 |
| TR54193\|c0_g1 | ---NA--- | 0,22 | 1,749 | 2 | 16 |
| TR4661\|c0_g1 | ---NA--- | 0,44 | 1,989 | 6 | 27 |
| TR54773\|c0_g1 | ---NA--- | 2,371 | 10,746 | 6 | 27 |
| TR20452\|c0_g1 | ---NA--- | 0,54 | 2,649 | 5 | 24 |
| TR56981\|c0_g1 | nudix hydrolase 8-like isoform x1 | 0,31 | 1,32 | 7 | 29 |
| TR36107\|c0_g1 | ---NA--- | 0,26 | 1,16 | 6 | 26 |
| TR50407\|c0_g1 | ---NA--- | 0,56 | 2,459 | 6 | 26 |
| TR46404\|c0_g1 | ---NA--- | 0,19 | 1,18 | 3 | 18 |
| TR52790\|c0_g1 | glutamine synthetase family protein | 0,45 | 2,749 | 3 | 18 |
| TR55002\|c0_g1 | ---NA--- | 0,42 | 2,549 | 3 | 18 |
| TR359\|c0_g1 | ---NA--- | 0,85 | 3,569 | 6 | 25 |
