# Supplementary material for: Comparative Transcriptome Reconstruction of Four Hypericum Species Focused on Hypericin Biosynthesis
Source: Front Plant Sci. 2016 Jul 13;7:1039. doi: 10.3389/fpls.2016.01039 (PMC4942478; doi:10.3389/fpls.2016.01039)
Supplement: Supplementary file 3 [file Table_3.DOCX]

| ***H. tomentosum* contig** | **NCBI-nr annotation** | **non-nodules**  **FPKM** | **nodules**  **FPKM** | **non-nodules**  **counts** | **nodules**  **counts** |
| --- | --- | --- | --- | --- | --- |
| TR38948\|c0_g1 | phenolic oxidative coupling protein | 0,185 | 46,474 | 3 | 550 |
| TR15166\|c0_g1 | polyketide synthase | 0,38 | 27,719 | 10 | 549 |
| TR18207\|c0_g1 | s-adenosyl-l-methionine-dependent methyltransferases superfamily | 0,226 | 18,697 | 7 | 427 |
| TR8620\|c0_g1 | phenolic oxidative coupling protein | 0 | 15,796 | 0 | 191 |
| TR39965\|c0_g1 | pollen ole e 1 allergen and extensin family | 16,069 | 361,435 | 304 | 5095 |
| TR9916\|c0_g1 | reticuline oxidase-like protein | 0,185 | 7,523 | 8 | 237 |
| TR44467\|c0_g1 | polygalacturonase inhibitor-like | 0 | 5,752 | 0 | 118 |
| TR5871\|c0_g1 | phenolic oxidative coupling protein | 1,428 | 27,719 | 28 | 405 |
| TR43006\|c0_g1 | polyketide synthase | 0,637 | 29,023 | 4 | 136 |
| TR22183\|c0_g1 | licodione synthase-like | 0,123 | 2,754 | 5 | 85 |
| TR43757\|c1_g1 | homogentisate chloroplastic-like | 1,664 | 13,091 | 39 | 289 |
| TR45204\|c0_g1 | glycosyl hydrolase family 1 family protein | 0,175 | 3,144 | 7 | 92 |
| TR1369\|c0_g1 | glutathione s-transferase | 0,123 | 4,302 | 2 | 52 |
| TR3869\|c0_g1 | truncated transcription factor cauliflower a-like | 0,051 | 3,027 | 1 | 40 |
| TR13640\|c0_g1 | udp-glycosyltransferase 76f1-like | 0 | 4,964 | 0 | 32 |
| TR45090\|c0_g1 | ---NA--- | 0,123 | 6,453 | 1 | 38 |
| TR45451\|c1_g1 | probable wrky transcription factor 72 isoform x1 | 0,257 | 2,9 | 7 | 60 |
| TR31690\|c0_g1 | chitinase 5 | 0 | 2,277 | 0 | 27 |
| TR15368\|c0_g1 | dirigent protein 22-like | 0,329 | 4,273 | 5 | 48 |
| TR3869\|c0_g2 | truncated transcription factor cauliflower a-like | 0,113 | 2,423 | 2 | 32 |
| TR25145\|c0_g1 | ugt protein isoform 1 | 0,329 | 2,842 | 9 | 58 |
| TR31055\|c0_g2 | isoform 1 | 2,661 | 13,723 | 92 | 354 |
| TR17341\|c0_g1 | vinorine synthase-like | 0,277 | 2,258 | 10 | 60 |
| TR28223\|c0_g1 | uncharacterized mitochondrial protein g00810-like | 5,343 | 25,003 | 248 | 865 |
| TR37845\|c0_g1 | protein srg1-like | 3,504 | 26,473 | 11 | 62 |
| TR48087\|c0_g1 | ubiquinone biosynthesis protein coq- | 0,123 | 1,771 | 3 | 32 |
| TR47058\|c0_g1 | ---NA--- | 0,411 | 7,446 | 2 | 27 |
| TR9916\|c0_g2 | reticuline oxidase-like protein | 0,072 | 2,706 | 1 | 27 |
| TR36299\|c0_g1 | non-specific lipid-transfer protein 1-like | 0,688 | 5,295 | 9 | 52 |
| TR54768\|c0_g1 | cytochrome p450 family protein | 0,298 | 3,124 | 5 | 39 |
| TR23105\|c0_g1 | ---NA--- | 1,099 | 8,088 | 10 | 55 |
| TR38177\|c0_g1 | Uncharacterized protein isoform 1 | 13,182 | 56,567 | 395 | 1263 |
| TR40330\|c0_g1 | PREDICTED: laccase-14-like | 0 | 3,611 | 0 | 20 |
| TR19610\|c0_g2 | dihydrofolate reductase-like protein | 0,956 | 5,548 | 21 | 91 |
| TR45409\|c0_g1 | high-affinity nitrate transporter family protein | 1,264 | 6,015 | 60 | 213 |
| TR32258\|c0_g1 | 2-oxoglutarate and fe -dependent oxygenase superfamily isoform 1 | 1,295 | 6,54 | 35 | 132 |
| TR51302\|c0_g1 | ---NA--- | 5,867 | 25,539 | 417 | 1300 |
| TR15723\|c0_g1 | ---NA--- | 1,079 | 9,626 | 6 | 40 |
| TR25753\|c0_g1 | protein srg1 | 5,291 | 29,023 | 23 | 94 |
| TR48913\|c0_g1 | Uncharacterized protein TCM_043094 | 0,154 | 1,956 | 3 | 28 |
| TR31305\|c0_g1 | alpha-xylosidase 2 | 8,908 | 36,011 | 491 | 1473 |
| TR10887\|c0_g1 | homogentisate chloroplastic | 0 | 1,168 | 0 | 17 |
| TR19610\|c0_g1 | dihydrofolate reductase-like protein | 1,603 | 7,251 | 37 | 125 |
| TR13161\|c0_g1 | glutathione s-transferase u17-like | 0 | 4,059 | 0 | 16 |
| TR18119\|c0_g1 | nac domain ipr003441 | 0,503 | 3,037 | 11 | 49 |
| TR50807\|c0_g1 | cystinosin homolog | 2,538 | 10,794 | 43 | 137 |
| TR49570\|c0_g1 | flavonol synthase flavanone 3-hydroxylase-like | 0,36 | 2,54 | 8 | 42 |
| TR32176\|c0_g1 | responsive to high light 41 family protein | 2,435 | 10,424 | 41 | 130 |
| TR46556\|c0_g1 | ---NA--- | 0,216 | 2,297 | 3 | 24 |
| TR10260\|c0_g1 | pathogenesis-related protein pr-4 | 1,778 | 8,682 | 22 | 80 |
| TR35846\|c0_g1 | cytochrome p450 cyp82d47-like | 2,425 | 18,434 | 6 | 34 |
| TR33298\|c0_g1 | calmodulin-binding protein of 25 | 0,257 | 2,667 | 3 | 23 |
| TR53858\|c0_g1 | PREDICTED: laccase-14-like | 0,298 | 2,667 | 4 | 27 |
| TR13113\|c0_g1 | nuclear transcription factor y subunit a-10 | 1,295 | 6,258 | 17 | 61 |
| TR28453\|c0_g1 | PREDICTED: uncharacterized protein LOC105973363 | 1,49 | 6,394 | 45 | 135 |
| TR22820\|c0_g1 | 0 | 0,164 | 1,577 | 3 | 22 |
| TR47529\|c0_g2 | phenolic oxidative coupling protein | 1,685 | 7,582 | 23 | 77 |
| TR15344\|c1_g5 | cystinosin homolog | 1,829 | 7,582 | 31 | 96 |
| TR36913\|c0_g1 | acidic mammalian chitinase-like | 0,175 | 2,122 | 2 | 18 |
| TR15503\|c0_g1 | ---NA--- | 0,288 | 1,956 | 6 | 30 |
| TR47794\|c0_g1 | ---NA--- | 0,267 | 1,801 | 6 | 30 |
| TR41071\|c0_g1 | PREDICTED: uncharacterized protein LOC105649794 isoform X1 | 1,428 | 9,285 | 6 | 29 |
| TR9339\|c0_g1 | ---NA--- | 0,843 | 6,482 | 4 | 23 |
| TR20361\|c0_g1 | ---NA--- | 0,432 | 2,686 | 6 | 28 |
| TR102\|c0_g2 | coatomer beta | 0,298 | 1,693 | 7 | 30 |
| TR30403\|c0_g1 | ---NA--- | 0,226 | 4,633 | 1 | 15 |
| TR38321\|c0_g1 | glutathione s-transferase u17-like | 0,247 | 4,866 | 1 | 15 |
| TR53287\|c0_g1 | wrky family transcription isoform partial | 0,432 | 2,813 | 5 | 24 |
| TR40011\|c0_g1 | brassinosteroid insensitive 1-associated receptor kinase 1 | 0,39 | 2,404 | 5 | 23 |
| TR19594\|c0_g1 | abscisic acid-insensitive 5-like protein 5 | 2,887 | 15,485 | 7 | 28 |
| TR30521\|c0_g1 | calmodulin-binding protein 25-like | 0,812 | 4,535 | 6 | 25 |
| TR52859\|c0_g1 | ---NA--- | 0,452 | 2,521 | 6 | 25 |
